# *Eucalyptus oleosa* Essential Oils: Chemical Composition and Antimicrobial and Antioxidant Activities of the Oils from Different Plant Parts (Stems, Leaves, Flowers and Fruits)

**DOI:** 10.3390/molecules16021695

**Published:** 2011-02-17

**Authors:** Hajer Naceur Ben Marzoug, Mehrez Romdhane, Ahmed Lebrihi, Florence Mathieu, François Couderc, Manef Abderraba, Mohamed Larbi Khouja, Jalloul Bouajila

**Affiliations:** 1 Laboratoire de Physicochimie des Matériaux, IPEST, BP51, La Marsa 2070, Tunisia; 2 Unité de Modélisation, Analyse et Commande des Systèmes, Ecole Nationale d’Ingénieur, 6029 Gabés, Tunisia; 3 Laboratoire Génie Chimique, UMR 5503 (CNRS/INPT/UPS), Université de Toulouse, INP/ENSAT, Avenue de l'Agrobiopole, BP 32 607, Auzeville-Tolosane, F-31326 Castanet-Tolosan Cedex, France; 4 LaboratoiredesInteractions Moléculaires et Réactivité Chimique et Photochimique, UMR CNRS 5623, Université de Toulouse, 118 route de Narbonne, F-31062 Toulouse, France; 5 Laboratoire d'Ecologie, INRGREF, B.P10, Ariana 2080, Tunisia

**Keywords:** *Eucalyptus oleosa* F. Muell. ex Miq., plant parts, essential oil, GC-MS, antioxidant activity, ABTS, DPPH, antimicrobial activity

## Abstract

Essential oils obtained by hydrodistillation from the different parts (stems, adult leaves, immature flowers and fruits) of *Eucalyptus oleosa* were screened for their antioxidant and antimicrobial properties and their chemical composition. According to GC-FID and GC-MS, the principal compound of the stem, immature flowers and the fruit oils was 1,8-cineole, representing 31.5%, 47.0% and 29.1%, respectively. Spathulenol (16.1%) and γ-eudesmol (15.0%) were the two principal compounds of adult leaves oil. In the DPPH (1,1-diphenyl-2-picrylhydrazyl) assay, the oils of the four parts showed moderate antioxidant activity. In the ABTS (2,2’-azinobis-3-ethylbenzothiazoline-6-sulphonate) assay, the most active part was the adult leaves, with a IC_50_ value 13.0 ± 0.6 mg/L, followed by stems (IC_50_ = 43.5 ± 1.4 mg/L). The essential oils showed a better antibacterial activity against Gram-positive and Gram-negative bacteria, and a significant antifungal activity also was observed against yeast-like fungi. A strong correlations between oxygenated monoterpenes and antimicrobial activity (especially 1,8-cineole) were noted (R^2^ = 0.99, 0.97 and 0.79 for *B. subtilis*, *P. aeruginosa* and *C. albicans*, respectively).

## Abbreviations

DPPH: 1,1-diphenyl-2-picrylhydrazyl
ABTS: 2,2’-azinobis-3-ethyl-benzothiazoline-6-sulphonate
IC_50_: half maximal inhibitory concentration
GC-FID: Gas Chromatography - Flame Ionization Detector
GC-MS: Gas Chromatography - Mass Spectrometry
KI: Kovats index
R^2^: Correlation coefficient

## 1. Introduction

The *Myrtaceae* family contains 133 genera and 3,800 species of trees and shrubs. This family occurs in temperate, subtropical, and tropical regions; however, it is centered in Australia, tropical to southern temperate America and Southeast Asia and has a little representation in Africa [1].

One of important genera of *Myrtaceae* is *Eucalyptus*,which is a large genus of evergreen trees and shrubs containing about 700 species [2]. Although most of the plants are native to Australia and Tasmania [3], they have been successfully introduced worldwide and are cultivated in many other countries, including Tunisia.

*Eucalyptus* trees are among the most important hard wood forestry crops worldwide and provide a major source of pulp wood for high quality paper production. *Eucalyptus* timber is used for construction and fuel [4], and *Eucalyptus* gum is used for diarrhoea and as an astringent in dentistry [2]. Moreover, *Eucalyptus* has been prized a rich source of essential oils. Essential oils of various species have been used in the pharmaceutical [5], cosmetics [6], food [4], industries and medicinal purposes [6,7,8]. The minimum 1,8-cineole content of pharmaceutical-grade *Eucalyptus* essential oil as defined in most standards is 70% [9]. In the cosmetics industry *Eucalyptus* essential oil is used in detergents [6], toiletries and little employed in perfumes and as a flavoring agent in food [10].

In addition, the essential oils of *Eucalyptus* species possesses important biological activities including diaphoretic, disinfectant, antimalarial, antiseptic, analgesic, antiinflammatory, antibacterial [11], expectorant [7] and antioxidant properties [12].

Many investigations have been done on the volatile components of essential oils from different species of *Eucalyptus*. Moreover, there are many reports which have determined the composition of essential oils from the leaves [13,14], immature flowers [8] and recently from the fruits [5]. Previous studies of the composition of *Eucalyptus oleosa* leaves essential oil have been reported [9,15,16], but to the best of our knowledge, no previous studies on the chemistry and antioxidant and antimicrobial activities of the essential oils of stem, immature flowers and fruit of *E. oleosa* have been presented in the literature to date. In the present paper the composition of the essential oils obtained separately from stems, adult leaves, immature flowers and fruits of *E. oleosa* accumulated in Tunisia south has been studied. We also investigated the antioxidant and antimicrobial activities of the essential oils. Correlations between chemical composition of essential oils and antimicrobial and antioxidant activities were studied. 

## 2. Results and Discussion

### 2.1. Chemical composition of the essential oils

The extraction yields of essential oils of the stems, adult leaves, fruits and immature flowers of *E. oleosa* were 0.52%, 0.45%, 1.12% and 0.53%, respectively (Table 1). In the literature [16], reported yields of oil from *E. oleosa* leaves from southern Tunisia collected in October 2007 was richer than our adult leaves harvested in March 2007, with a variation of 4.45%. *E. oleosa* from the center of Tunisia [15] was richer in the essential oil than that of south with a difference of 2.25%. The yield variability in the total essential oil of *E. oleosa* species growing in Tunisia can be attributed to the soil conditions and ecological, climatic conditions, age of plant and the season of harvest.

**Table 1:** Chemical composition of the different parts of *E. oleosa* essential oil.

Peak	KI	Compounds	Stems	Adult leaves	Fruits	Immature flowers
1	936	α-Pinene	5.2	1.7	2.6	2.2
2	951	α-Fenchene	1.9			0.0
3	954	Camphene	0.4	3.4	0.5	0.2
4	976	Sabinene			0.2	0.6
5	1025	*p*-Cymene	6.8	10.6	9.0	9.2
6	1028	Limonene	4.2	1.5	0.7	1.6
7	1030	1,8-Cineole	31.5	8.7	29.1	47.0
8	1033	β-Phellandrene	0.2			0.1
9	1057	γ-Terpinene	0.4	0.7	0.4	0.5
10	1086	α-Terpinolene	0.3	0.2	0.1	0.2
11	1091	Methyl benzoate				0.3
12	1108	*endo*-Fenchol			0.1	0.2
13	1137	4-Acetyl-1-methylcyclohexene		1.1	0.6	0.4
14	1138	*trans*-2-Caren-4-ol*		0.7	0.2	0.2
15	1140	*cis*-Sabinol	3.1	4.2	2.5	1.0
16	1141	*trans*-Pinocarveol	9.9		0.1	0.1
17	1166	Borneol	0.9	0.5	0.3	1.1
18	1168	Pinocarvone	3.5	1.8	1.0	0.3
19	1185	*p*-Cymen-8-ol	1.9	4.4	1.2	1.4
20	1195	Myrtenal	0.3	1.0	0.3	0.2
21	1202	Myrtenol	0.5	0.7	0.3	0.2
22	1208	Verbenone	2.1	3.7	1.4	0.8
23	1231	*cis*-Carveol			0.1	0.1
24	1235	Neral			0.1	0.1
25	1237	Pulegone	0.3	0.2	0.1	0.3
26	1237	Cuminaldehyde	2.2	3.1	0.9	1.2
27	1280	Piperitone	0.4		0.3	0.1
28	1286	Bornyl acetate	1.1			0.0
29	1288	*p*-Cymen-7-ol	1.1	4.0	0.8	0.8
30	1302	Carvacrol		1.6	0.4	0.4
31	1320	Methyl geranate	0.6		0.1	0.2
32	1340	Methyl 2-methoxybenzoate*				0.1
33	1354	*exo*-2-Hydroxycineole acetate*	0.3		0.1	0.2
34	1373	Isoledene*			0.3	0.1
35	1380	α-Copaene*			0.2	0.1
36	1388	β-Bourbonene	0.4		1.7	0.4
37	1404	Methyl eugenol	0.3			0.0
38	1407	α-Gurjunene			0.2	0.5
39	1456	γ-Selinene	0.4		0.5	0.3
40	1480	γ-Muurolene			0.6	0.3
41	1487	β-Selinene			1.3	1.1
42	1493	Viridiflorene			0.3	0.6
43	1516	α-Selinene	0.5		10.0	2.1
44	1526	δ-Cadinene		0.8	1.2	1.4
45	1527	Calamenene	0.4	0.3	0.5	0.4
46	1565	Ledol		1.0	1.2	1.0
47	1576	Spathulenol	3.5	16.1	3.4	0.0
48	1578	Globulol	0.4	0.7	0.9	0.7
49	1580	β-Caryophyllene oxide	0.5	1.1	0.7	0.8
50	1590	Viridiflorol	0.6	1.1	2.3	1.4
51	1596	Guaiol	1.6	2.8		0.0
52	1620	(+)-Spathulenol		0.6		0.2
53	1648	γ-Eudesmol	5.6	15.0	16.4	12.5
54	1650	α-Cadinol	1.0	2.0	3.3	2.3
55	1651	β-Eudesmol	3.2	1.0		0.0
56	1805	Nootkatone*	0.3	0.5	0.5	0.3
57	nd	Dihydroumbellulone	0.5	0.4	0.2	0.3
58	nd	Methyl ionone*		0.1	0.1	0.2
59	nd	5,7-Dimethyl-1-tetralone*	0.4	0.6	0.3	0.3
60	nd	trans-Longipinocarveol*		0.4		0.0
61	nd	Platambin*		0.7	0.3	0.1
Total			98.8	99.1	99.6	99.1
Monoterpene hydrocarbons			12.6	7.5	4.5	5.5
Monoterpenes Oxygenated			52.9	21.2	35.9	51.9
Sesquiterpenes hydrocarbons			1.8	1.1	16.7	7.3
Sesquiterpenes Oxygenated			16.7	43.2	29.0	19.4
Others			14.9	24.6	13.1	14.6
Phenolics			0.0	1.6	0.4	0.4
Extraction yields (%)			0.52	0.45	1.12	0.53

^* ^Tentatively identified according to the mass spectrum (MS) and by comparison of KI with the literature. nd: not determined.

**Figure 1:**
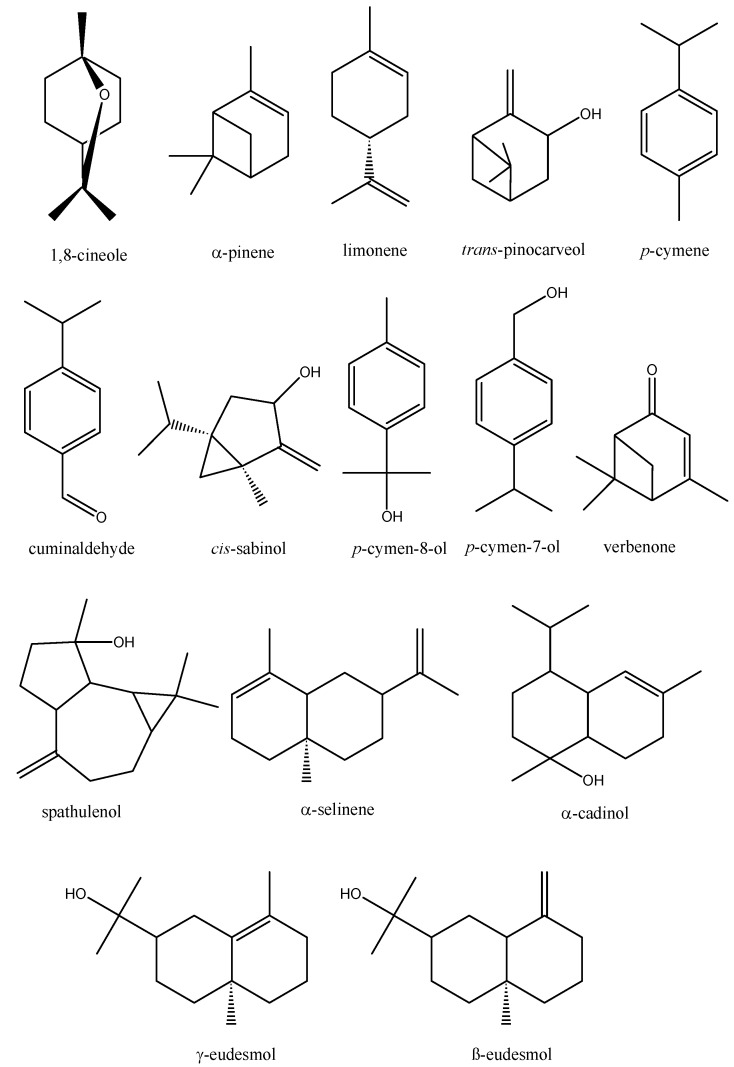
Structures of the most abundant compounds identified in *E. oleosa* essential oils*.*

Table 1 shows the identified constituents, the percentage composition and their Kovats index (KI) values obtained on a DB-5MS capillary column. The main constituents of all *E. oleosa* essential oils are presented in Figure 1.

In the essential oil extracted from *E.* *oleosa* stems 40 compounds were identified, corresponding to 98.8% of the total essential oil. The essential oil of stems contained 84.0% of terpene derivatives (65.5% and 18.5% monoterpenes and sesquiterpenes, respectively). Oxygenated monoterpenes (52.9%) were prevalent compared to monoterpenes hydrocarbons (12.6%). Moreover among sesquiterpenes, the oxygenated species were also detected in a higher percentage (16.7%) than hydrocarbons (1.8%). The main constituents (Figure 1) in the *E. oleosa* stems essential oil were 1,8-cineole (31.5%), *trans*-pinocarveol (9.9%), *p-*cymene (6.8%), γ-eudesmol (5.6%), α-pinene (5.2%), limonene (4.2%), pinocarvone (3.5%) and spathulenol (3.5%).

Thirty eight compounds amounting to 99.1% were identified in *E. oleosa* leaf essential oil. The essential oil from adult leaves contained about twice the amount of sesquiterpenes than monoterpenes (44.3%, 28.7% respectively). Spathulenol (16.1%) and γ-eudesmol (25%) were the main oxygenated sesquiterpenes in leaf essential oil, followed by *p-*cymene (10.6%), 1,8-cineole (8.7%), *p-*cymen-8-ol (4.4%), cis-sabinol (4.2%), *p-*cymen-7-ol (4.0%) and verbenone (3.7%).

In the essential oil of the fruits, 51 compounds were identified. The sesquiterpenes constituted 45.7% and the monoterpenes constituted 40.4%, of which the oxygenated monoterpenes had the most important contributions (35.9%). 1,8-Cineole (29.1%), γ-eudesmol (16.4%), α-selinene (10.0%) and *p-*cymene (9.0%) were the major compounds in fruits essential oil.

The essential oil of immature flowers was constituted by merely 54 compounds. This essential oil showed a higher percentage of monoterpenes (57.4%) compared to sesquiterpenes (26.7%). Oxygenated monoterpenes were the most important derivatives identified, mainly because of the high percentage of 1,8-cineole (47.0%). The other main compounds identified in the essential oil of immature flowers were γ-eudesmol (12.5%) and *p-*cymene (9.2%).

Comparing the four essential oils, the highest amounts of monoterpenes were found in stems (65.5%), followed by immature flowers (57.4%) and fruits (40.4%) and the lowest amount was in adult leaves (28.7%). Stems and immature flowers were the main producers of oxygenated monoterpenes (52.9% and 51.9%, respectively). For sesquiterpenes, the highest percentages were detected in similar amounts in fruits (45.7%) and adult leaves (44.3%), followed by immature flowers (26.7%) and stems (18.5%). The highest value of oxygenated derivatives was found in adult leaves (43.2%).

The principal differences in the four essential oils were related to 1,8-cineole, spathulenol and γ-eudesmol: the highest percentage of 1,8-cineole (47.0%) was detected in essential oil from immature flowers, whereas it was lowest in adult leaves (8.7%). Spathulenol found in essential oil from adult leaves (16.1%) was about four times that produced by stems (3.5%) and fruits (3.4%), whereas it not detected in immature flower essential oils. In the case of γ-eudesmol, the most important percentage was found in fruits (16.4%), followed by adult leaves (15.0%) and immature flowers (12.5%) and it was detected with a lowest percentage in the stems (5.6%).

The chemical composition of *E. oleosa* essential oils had been reported before [9,15,16], but the data analysis shows that the chemical composition differs according to the origin of the plants. There are no reports on chemical composition of essential oils obtained from stems, immature flowers and fruits of*E. oleosa*.

The chemical composition of essential oil of leaves of *E. oleosa* grown in south Tunisia and collected in autumn was reported in [16]. In this work, we studied the oil obtained from adult leaves collected in March 2007 (Spring). Comparing the two chemical compositions we noted a significant variation. α-Pinene (21.8%), 1,8-cineole (41.2%), *trans*-pinocarveol (5.7%), *p-*cymene (3.3%), limonene (3.1%) and γ-eudesmol (5.3%) were the major compounds of leaves of the autumnal collection of *E. oleosa*, while the main compounds of the spring adult leaves were spathulenol (16.1%) and γ-eudesmol (15.0%). The percentage of α-pinene and 1,8-cineole contained in our adult leaves of *E. oleosa* was lower than that of the leaves collected in October 2007. Spathulenol was not found in the essential oil of *E. oleosa* harvested in Autumn. Moreover, the percentage of γ-eudesmol (5.3%) contained in leaves essential oil of *E. oleosa* collected in October 2007 was lower than our adult leaves. The observed difference may be due to the seasonal variation and to the age of the leaves.

Comparing the chemical compositions *E. oleosa* growing in the center of Tunisia [15] to our work, important variations were noted. α-Pinene (12.3%), limonene (12.1%) and 1,8-cineole (26.1%) were the major compounds. We note that the percentage of 1,8-cineole was higher than ours. Moreover, the proportions of *p-*cymene, *p*-cymen-8-ol and spathulenol of adult leaf of *E. oleosa* from the south were more important than these of the center. *cis*-Sabinol, verbenone, cuminalaldehyde, *p-*cymen-7-ol, guaiol, α-cadinol and γ-eudesmol were present in our essential oil and had not been previously found in the essential oil of *E. oleosa* from the center of Tunisia.

The *E*. *oleosa* essential oil from western Australia was reported by Barton *et al.* [9] to contain α-pinene (2%), *p*-cymene (2.5%), 1,8-cineole (75.5%). A remarkable difference was also found between our work and that of Barton *et al*. The Australian *E*. *oleosa* essential oil showed a higher percentage of oxygenated monoterpenes, mainly because of the important percentage of 1,8-cineole (75.5%). Oxygenated sesquiterpenes are predominant in our essential oil, because of the remarkable proportions of spathulenol (16.1%) and γ-eudesmol (15.0%). Moreover, cis-sabinol, *p-*cymen-8-ol, verbenone, cuminalaldehyde, *p-*cymen-7-ol, spathulenol, guaiol, γ-eudesmol and α-cadinol were not found in the essential oil of *E*. *oleosa* from Australia.

We thus note significant differences in the chemical composition of *E*. *oleosa* leaf essential oil native to the center or south of Tunisia and in Australia. These qualitative and quantitative differences in the chemical composition of essential oils could be attributed to several factors such as geographical location, climatic effects of the plants, harvest season, nature of the soil, age of the plant parts (young or adult), the state of plant material used (dried or fresh), the part of the plant used, time of collection, *etc*.

### 2.2. Antioxidant activity

Antioxidant activity of essential oils extracted by hydrodistillation from stems, adult leaves, immature flowers and fruits of *E*. *oleosa* has been determined by two different test systems, namely, the DPPH and ABTS assays. All data are presented in Table 2.

**Table 2:** Antioxidant activity (IC_50_ (mg/L)) of the different parts of *E. oleosa* essential oil.

Samples	DPPH assay	ABTS assay
Stems	> 10000	43.5 ± 1.4
Adult leaves	1536.3 ± 40.5	13.0 ± 0.6
Fruits	441.1 ± 12.7	51.0 ± 1.3
Immature flowers	1270.4 ± 33.4	79.3 ± 1.9
vit C	4.4 ± 0.2	1.9 ± 0.1

According to the DPPH assay results, the essential oils of the four parts of *E*. *oleosa* showed moderate antioxidant activity. The most active plant part was the fruit, with an IC_50_ value of 441.1 ± 12.7mg/L, followed by immature flowers (IC_50_ = 1270.4 ± 33.4 mg/L).

In the ABTS assay, the best IC_50_ was found for the adult leaves essential oil (13.0 ± 0.6 mg/L). This activity was followed by stem essential oil (IC_50_ = 43.5 ± 1.4 mg/L), fruits essential oil (IC_50_ = 51.0 ± 1.3 mg/L) and immature flowers essential oil (IC_50_ = 79.3 ± 1.9 mg/L). This result was different compared with that of DPPH assay. In fact, this important activity compared to other samples was due to the specificity of ABTS radical cation. The antioxidant activity of stems, immature flowers and fruits essential oils of *E*. *oleosa* has not been reported in the literature.

The antioxidant activity of α-pinene [21] and *p*-cymene [12] were previously reported and they did not show appreciable antioxidant activity. According to previous reports, γ-terpinene has shown a high activity [20]. The antioxidant activity of limonene was previously reported [21] and determined by ferric reducing antioxidant power (FRAP) method; it has an important reducing ability (37.440 mmol/ kg). The moderate antioxidant activity is probably due to the low amount of phenolic compounds present. No correlation was obtained between the antioxidant activity by DPPH and ABTS and chemical composition. 

### 2.3. Antimicrobial activity

The antimicrobial activity of essential oils from different parts (stems, adult leaves, fruits and immature flowers) of *E*. *oleosa* were tested at various concentrations (0.5–20 mg/mL) and their antimicrobial potency was assessed by MIC values (Table 3). The results showed that the essential oil of all the plant parts of *E*. *oleosa* had great potential antimicrobial activity against all microorganisms. As shown in Table 3, all parts (stems, adult leaves, fruits and immature flowers) of *E*. *oleosa* exhibited antibacterial activity, although the immature flowers presented a larger prevalence of activity (0.93–3.72 mg/mL). The strongest antifungal activity was observed using the essential oil from *E*. *oleosa* immature flowers and stems, with MIC values between 2.79–3.88 mg/mL.

Essential oil of different parts appeared more active against the tested Gram-positive than Gram-negative bacteria. This result was in agreement with many studies realized on other plant species like *E. robusta, E. alba, E. camadulensis, E. citriodora, E. globulus, E. saligna* [11]. High resistance among Gram-negative bacteria could be due to the differences in the cell membrane of these bacterial groups. Gram-negative bacteria possess an outer membrane surrounding the cell wall, which restricts diffusion of hydrophobic compounds through its lipopolysaccharide covering. Without this barrier, the membrane in Gram-positive bacteria can be permeated more easily and disrupt the proton motive force, electron flow, active transport, and coagulation of the cell contents [22,23].

The antimicrobial activity of stem, immature flower and fruit essential oils from *E. oleosa* has not been reported elsewhere. Leaves essential oils of *E. oleosa* growing in south Tunisia and collected in October 2007 [16] exhibited an interesting antimicrobial activity against all microorganisms tested except *S.aureus* and *E*. *coli*. This significant difference is probably due to the time of harvest and the age of the samples.

The antimicrobial properties of essential oils from different parts of *E*. *oleosa* are suspected to be associated with their high contents of oxygenated compounds (1,8-cineole, *p-*cymene, sphathulenol, γ-eudesmol). According to Nada *et al.* [24] and to Tabanca *et al.* [25], 1,8-cineole, sphathulenol, *p-*cymene and α-pinene have significant antimicrobial activity. Other compounds as carvacrol, γ-terpinene, borneol, limonene, myrtenal, cuminaldehyde and camphene which are a minor constituents in our essential oils are also known to have efficient antimicrobial properties [11,22,24].

**Table 3:** Antimicrobial activity (MIC) of the different parts of *E. oleosa* essential oil.

The minimal inhibitory concentration (MIC (mg/mL))												
	Gram-positive bacteria			Gram-negative bacteria				Yeast		Fungi		
Samples	*B. subtilis*	*S. aureus*	*L. monocytogenes*	*P. aeruginosa*	*S. enterica*	*E. coli*	*K. pneumoniae*	*S. cerevisiae*	*C. albicans*	*A. ochraceus*	*M. ramamnianus*	*F. culmorum*
Stems	1.94	1.94	1.94	2.91	2.91	1.94	2.91	2.91	2.91	3.88	3.88	3.88
Adult leaves	4.65	1.86	1.86	6.51	2.79	2.79	4.65	4.65	4.65	4.65	6.51	4.65
Immature flowers	0.93	1.86	1.86	2.79	1.86	1.86	1.86	2.79	2.79	2.79	2.79	3.72
Fruits	4	2.4	2.4	6	3.2	3.2	2.4	3.2	4	6	6	3.2
Ampicillin	0.02	0.02	0.02	0.005	0.02	0.02	0.01	0.015	0.015	0.02	0.02	0.02
Nalidixic acid	0.02	0.02	0.02	0.013	0.02	0.02	0.013	0.008	0.02	0.02	0.02	0.02
Nystatin	0.02	0.008	0.008	0.02	0.02	0.02	0.015	0.02	0.02	0.01	0.02	0.015

A chemical composition and antimicrobial activity relationship deduced from our results has demonstrated that the antimicrobial activity of the studied different organs studied was related to the presence of oxygenated terpenes. For *B. subtilis* (Gram positive), the oxygenated terpenes (R^2^ = 0.99) and especially oxygenated monoterpenes (R^2^ = 0.87) were responsible for activity against this strain. Furthermore, the content of oxygenated monoterpenes in the different essential oils were correlated with their antibacterial activity, confirming that oxygenated monoterpenes were likely major contributors to the antibacterial activity against *P. aeruginosa* (R^2^ = 0.91) and *K. pneumoniae* (R^2^ = 0.63). They were also responsible for the antifungal activity against *C. albicans* (R^2^ = 0.98), *S. cerevisiae* (R^2^ = 0.88) and *M. ramamnianus* (R^2^ = 0.85).

In conclusion, the correlations between the chemical composition of the here studied essential oils and the antimicrobial activity is related to the presence of oxygenated compounds and especially 1,8-cineole. It presented an interesting correlation with a coefficient R^2^ = 0.99, 0.97, 0.79 and 0.78 for *B. subtilis*, *P. aeruginosa C. albicans* and *M. ramamnianus*, respectively. 

Our study can be considered as the first detailed report on the chemical composition, antioxidant and antimicrobial activity of the essential oils extracted from stems, adult leaves, immature flowers and fruits of *E. oleosa.* The results presented here suggest that the essential oils obtained from different parts of *E. oleosa* possess antioxidant and antimicrobial properties and possess important percentages of 1,8-cineole and therefore, they could be used as preservative ingredients in the food and pharmaceutical industry.

## 3. Experimental

### 3.1. Plant materials

Aerial parts of *E. oleosa* were collected in south Tunisia (Gabés) in March 2007. The stems, adult leaves, immature flowers and fruits were separated by hand. After air-drying, the plant material was subject to hydrodistillation using a Clevenger-type apparatus. Specimens were identified in the Regional Station of the National Institute of Research in Farming Science, Waters and Forests. The arboretum was established in April 1960, the seed is of Australian origin.

### 3.2. Isolation of essential oils

Dried aerial parts (stems, adult leaves, immature flowers and fruits) of the plants (80 g) were separately ground, and submitted to hydrodistillation for 3 h using a Clevenger-type apparatus according to the method recommended by the European Pharmacopoeia (1983). For each sample, two replicate extractions were done. The obtained essential oils were dried over anhydrous sodium sulphate and after filtration stored at 4 °C until tested and analysed without further extraction. Yields are shown in Table 1.

### 3.3. Chemicals

All chemicals used were of analytical reagent grade. All reagents were purchased from Sigma-Aldrich-Fluka (Saint-Quentin, France).

### 3.4. Gas chromatography and gas chromatography-mass spectrometry

#### 3.4.1. Gas chromatography

Essential oils were analyzed using a Varian Star 3400 (Les Ulis, France) Cx gas chromatograph equipped with a flame ionization detection (FID) detector and DB-5MS capillary column (30 m × 0.25 mm, film thickness 0.25 μm). Injector and detector temperatures were set at 200 °C and detector temperature 270 °C, respectively. Oven temperature gradually raised from 60 °C to 260 °C at 5 °C /min, held for 15 min and finally raised to 340 °C at 40 °C /min. Helium (purity 99.99999%) was the carrier gas, at a flow rate of 1 mL/min. Total analysis time was 57 min. Diluted sample (1/100 in petroleum ether, v/v) of 1.0 µL was injected in the split mode (ratio 1:10). Quantitative data were obtained electronically from FID area percent data without the use of correction factors. Peak integration and quantification were performed automatically with Saturn 2100 Workstation software. A checking of the integration of each peak is carried out and corrected manually if necessary.

#### 3.4.2. Gas chromatography/mass spectrometry (GC-MS)

Analysis of essential oils was performed under the same conditions with GC (column, oven temperature, flow rate of the carrier gas) using a Varian Star 3400 (Les Ulis, France) gas chromatograph equipped with a Varian Saturn GC/MS/MS 4D mass selective detector in the electron impact mode (70 eV). Injector and MS transfer line temperatures were set at 200 and 300 °C, respectively. MS was adjusted with an emission current of 10 µA and electron multiplier voltage at 1500 V. Trap temperature was 250 °C and mass scanning was from 40 to 650 amu.

The components were identified based on the comparison of their Kovats indices (KI), co-injection of standards, MS experimental data with those contained in commercial or literature libraries (NIST 02 version 2.62, Adams, 2001). Alkanes (C_5_-C_24_) were used as reference points in the calculation of KI. GC and GC-MS analysis results are given in Table 1. All determinations were performed in duplicate and averaged.

### 3.5. Antioxidant activity

#### 3.5.1. DPPH Radical scavenging assay

Antioxidant scavenging activity was studied using 1,1-diphenyl-2-picrylhydrazyl free radical (DPPH) as described by Blois [17] with some modifications. Various dilutions of the test materials (pure antioxidant or essential oils, 1.5 mL) were mixed with a 0.2 mM methanolic DPPH solution (1.5 mL). After an incubation period of 30 min at 25 °C, the absorbance at 520 nm (wavelength of maximum absorbance of DPPH^·^) were recorded as A_(__sample)_, using a Helios spectrophotometer (Unicam, Cambridge, UK). A blank experiment was also carried out applying the same procedure to a solution without the test material and the absorbance was recorded as A_ (blank)_. The free radical-scavenging activity of each solution was then calculated as percent inhibition according to the following equation:

%inhibition = 100 (A_ (blank)_ – A_ (sample)_) / A_ (blank)_

Antioxidant activity of test compounds or extracts was expressed as IC_50_, defined as the concentration of the test material required to cause a 50% decrease in initial DPPH^·^ concentration. All measurements were performed in triplicate.

#### 3.5.2. ABTS radical-scavenging assay

The radical scavenging capacity of antioxidant for the ABTS (2,2’-azinobis-3-ethylbenzothiazoline-6-sulphonate) radical action was determined as described by Re *et al.* [18]. ABTS was generated by mixing 7 mM of ABTS at pH 7.4 (5 mM NaH_2_PO_4_, 5 mM Na_2_HPO_4_ and 154 mM NaCl) with 2.5 mM potassium persulfate (final concentration) followed by storage in the dark at room temperature for 16 h before use. The mixture was diluted with ethanol to give an absorbance of 0.70 ± 0.02 units at 734 nm using spectrophotometer (Helios, Unicam, Cambridge UK). For each sample, the diluted methanol solution of essential oil (100 μL) was allowed to react with fresh ABTS solution (900 μL), and then the absorbance was measured 6 min after initial mixing. Ascorbic acid was used as a standard. The capacity of free radical scavenging was expressed by IC_50_ (mg/L) value, wich represents the concentration required to scavenge 50% of ABTS radicals. The capacity of free radical scavenging IC_50_ was determined using the same equation previously used for the DPPH method. All measurements were performed in triplicate.

### 3.6. Microbial strains

All strains were obtained from the Laboratory of Chemical Engineering, Bioprocess Systems Microbial Department, Ecole Nationale Supérieure Agronomique de Toulouse. The different essential oils were individually tested against a panel of microorganisms. Seven bacteria including three Gram-positive (*Staphylococcus aureus* CIP 7625, *Listeria monocytogenes* Scott A 724, *Bacillus subtillis* ATCC 6633), and four Gram-negative bacteria (*Pseudomonas aeruginosa CIPA22, Salmonella enterica CIP833, Escherchia coli* ATCC 10536, *Klebseilla pneumoniae* CIP 8291) were used. Two yeasts **(***Saccharomyces cerevisiae* EDV 436 and *Candida albicans* IPA 200) and three fungi (*Aspergillus parasiticus* NRRL 3174, *Mucor ramamnianus* NRLL 1829 and *Fusarium culmorum* NRRL 3288) were also tested. The bacterial and yeast strains were cultured on nutrient agar for 48 h at 37 °C, while fungi were propagated in PDA (*Fusarium culmorum*) and in ISPD_2_ (*Aspergillus parasiticus*, *Mucor ramamnianus*) at 30 °C for 48 h to 3 days before used. All microorganisms were stocked at −6 °C in appropriate conditions and regenerated twice before use in the manipulations.

### 3.7. MIC agar dilution assay

The minimal inhibition concentration (MIC) values of microorganisms were studied, based on the agar dilution method [19]. The essential oils of different parts of *E. oleosa* were dissolved with methanol (400 μL essential oil in 400 μL methanol). The essential oils dissolved were added aseptically to sterile nutrient agar supplemented with Tween 80 (0.5%) at appropriate volume to produce the concentration range of 0.5–20 mg/mL.

The resulting nutrient agar solutions were immediately poured into petri dish after vortexing and allowed to solidify. The dish was left to cool down and to solidify at room temperature for 30 min. The plates were spotted, then inoculated with 1 μL of bacterial strains (4 × 10^6^ cells/mL) and of yeast and fungi (25 × 10^5^ spores/mL). Tests were carried out in duplicate.

Ampicillin and nalidixic acid (0.5–20 mg/L) were used as positive reference standards to determine the sensitivity of Gram-positive and Gram-negative bacterial species, respectively, tested. Nystatin (0.5–20 mg/L) was used as a positive reference standard to determine the sensitivity of fungi and yeast species. These plates, after staying at 4 °C for 2 h, were incubated at 37 °C for bacteria and at 30 °C for 48 h for yeast and fungi. Tests were carried out in duplicate.

### 3.8. Statistical analysis

All data of antioxidant activity were expressed as means ± standard deviations (SD) of triplicate measurements. The confidence limits were set at P < 0.05. SD did not exceed 5% for the majority of the values obtained. Correlation coefficients (R^2^) to determine the relationship between chemical composition and antioxidant or biological activity were calculated using MS Excel software (CORREL statistical function).

## 4. Conclusions

The results of the present study have revealed that the chemical composition and antioxidant and antimicrobial activity of essential oils of *E. oleosa* species, varied greatly depending upon the different parts of the plants extracted.

The antimicrobial activity of essential oils from different parts of *E. oleosa* may be directly associated with their major constituents or the presence of synergy between the major and minor constituents within the oil. As a result of this study, we will test the individual compound for their possible antimicrobial effects.

The relationship evaluated between the chemical composition and the antimicrobial activity suggested that the oxygenated groups were the most important factor in determining the antimicrobial activity (for example R^2^ = 0.99 for *B. subtilis* and *M. ramamnianus*). According to our results, 1,8-cineole was the principal compound responsible for the antibacterial and antifungal activities, its percentage was lower in adult leaves (8.7%) when the lowest antimicrobial activity has been observed in their essential oil.
